# Different Brain Activation under Left and Right Ventricular Stimulation: An fMRI Study in Anesthetized Rats

**DOI:** 10.1371/journal.pone.0056990

**Published:** 2013-02-22

**Authors:** Hideaki Suzuki, Akira Sumiyoshi, Ryuta Kawashima, Hiroaki Shimokawa

**Affiliations:** 1 Department of Cardiovascular Medicine, Tohoku University Graduate School of Medicine, Sendai, Japan; 2 Department of Functional Brain Imaging, Institute of Development, Aging and Cancer, Tohoku University, Sendai, Japan; 3 Division of Developmental Cognitive Neuroscience Institute of Development, Aging and Cancer, Tohoku University, Sendai, Japan; 4 Department of Advanced Brain Science, Institute of Development, Aging and Cancer, Tohoku University, Sendai, Japan; Tokyo Metropolitan Institute of Medical Science, Japan

## Abstract

**Background:**

Myocardial ischemia in the anterior wall of the left ventricule (LV) and in the inferior wall and/or right ventricle (RV) shows different manifestations that can be explained by the different innervations of cardiac afferent nerves. However, it remains unclear whether information from different areas of the heart, such as the LV and RV, are differently processed in the brain. In this study, we investigated the brain regions that process information from the LV or RV using cardiac electrical stimulation and functional magnetic resonance imaging (fMRI) in anesthetized rats because the combination of these two approaches cannot be used in humans.

**Methodology/Principal Findings:**

An electrical stimulation catheter was inserted into the LV or RV (n = 12 each). Brain fMRI scans were recorded during LV or RV stimulation (9 Hz and 0.3 ms width) over 10 blocks consisting of alternating periods of 2 mA for 30 sec followed by 0.2 mA for 60 sec. The validity of fMRI signals was confirmed by first and second-level analyses and temporal profiles. Increases in fMRI signals were observed in the anterior cingulate cortex and the right somatosensory cortex under LV stimulation. In contrast, RV stimulation activated the right somatosensory cortex, which was identified more anteriorly compared with LV stimulation but did not activate the anterior cingulate cortex.

**Conclusion/Significance:**

This study provides the first evidence for differences in brain activation under LV and RV stimulation. These different brain processes may be associated with different clinical manifestations between anterior wall and inferoposterior wall and/or RV myocardial ischemia.

## Introduction

It is empirically known that different clinical manifestations are observed in myocardial ischemia of the anterior wall of the left ventricle (LV) and in myocardial ischemia of the inferior wall and/or the right ventricle (RV). Patients with anterior wall myocardial ischemia typically present with angina and increased blood pressure and heart rate, whereas atypical symptoms, such as nausea, vomiting and decreased blood pressure and heart rate, occur more commonly in patients with inferior wall and/or RV myocardial ischemia [1]–[4]. Moreover, intracoronary thrombolytic therapy for occlusion of the right coronary artery, which supplies the inferior wall and RV, exhibits a greater incidence of bradycardia and hypotension than therapy for the occlusion of the left coronary artery, which affects the anterior wall [4]. These different manifestations can be explained by the different innervation of cardiac afferent nerves between the anterior wall and the inferior wall or RV. Afferent fibers to dorsal root ganglia (sympathetic afferents) predominantly innervate the anterior wall, whereas the fibers to nodose ganglia (vagal afferents) are concentrated in the inferior wall [1], [5], [6]. Vagal afferents are also predominantly innervated in the RV, although there are fewer afferents in the RV than in the LV [2]. These two types of cardiac afferent nerves may transmit information from the heart to different brain regions, thus causing different manifestations for anterior wall myocardial ischemia and inferior wall and/or RV myocardial ischemia. However, it remains unclear whether information from different heart areas, such as LV and RV, is processed differently in the brain.

Electrical stimulation is an effective means of selectively stimulating organs, including the heart. We have recently demonstrated that cerebral evoked potentials (CEPs) are induced in humans by increasing the intensity of cardiac pacing stimulation [7]. Electroencephalography (EEG) data, including CEPs, are direct measures of neuronal activity and are observed with high temporal resolution but poor spatial resolution [8]. In contrast, functional magnetic resonance imaging (fMRI) is a powerful tool for mapping activated brain regions with high spatial resolution [8] but is generally contraindicated for the use in patients implanted with cardiac pacing devices due to safety concerns, such as heat generation, arrhythmias, and device malfunction [9], [10]. In the present study, we investigated the brain regions that process information from the LV and RV using cardiac electrical stimulation and fMRI in anesthetized rats. Rats are a useful species for fMRI experiments and have been used to investigate the neural substrates of forepaw stimulation [11], [12], visual stimulation [13]–[16], auditory stimulation [17], [18], rectal distention [19], [20], appetite [21], gut administration of nutrition [22]–[26], pancreatic inflammation [27], and gustatory stimulation [28]. To minimize the hemodynamic effects of cardiac pacing, cardiac electrical stimulation was administered in a stepwise manner, as was performed in the previous CEP study in humans [7]. The validity of step-wise increases in stimulation intensity during an fMRI study was confirmed in an experiment that investigated fMRI signal changes during the well-established forepaw electrical stimulation [11], [12]. The hypothesis is that changes in fMRI signals are observed in different brain regions under LV and RV stimulation respectively.

## Results

### Validation of Step-wise Increase in Stimulation Intensity during an fMRI Study

**Figure 1:**
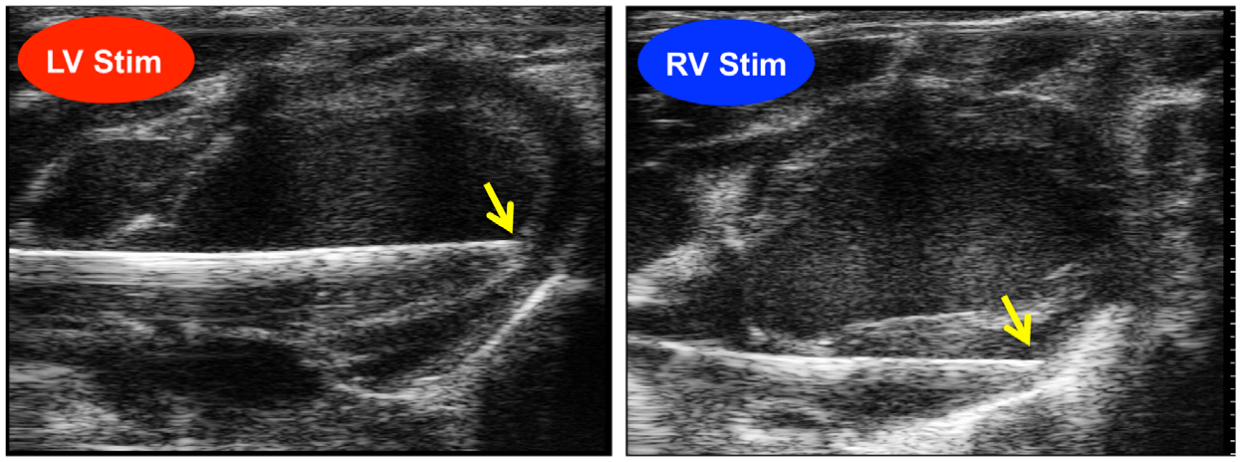
Representative echocardiograms of the electrical stimulation catheter inside the left (LV) or right ventricle (RV). Yellow arrowheads indicate the tip of the catheter from which electrical pulses were administered. The tip of the catheter was positioned at the interventricular septum in LV and the free wall in RV.

For selectively stimulating the heart, an electrical stimulation catheter was inserted into either the LV or RV (**Figure 1**). The measured cardiac pacing threshold and heart rate (HR) were 0.03–0.11 mA and 6–8.5 Hz (360–510 beats/min), respectively; the stimulation parameters of 0.2 mA, 9 Hz, and 0.3 msec width were chosen from these ranges to ensure continuous cardiac pacing.

To assess fMRI signals under LV or RV stimulation, step-wise increases in stimulation intensity from 0.2 to 2 mA were used in the present study. Electrical stimulation of the heart could induce sudden hemodynamic changes, which are likely to influence both brain activation and fMRI signals [29]–[34]. Actually, the initiation of LV or RV pacing with an intensity of 0.2 mA induced sudden changes in mean arterial blood pressure (mABP) and HR (**Figure S1 A, C**). In contrast, increasing the LV or RV stimulation intensity from 0.2 to 2 mA did not cause sudden or considerable changes in mABP (±2 mmHg) or HR (**Figure S1 B, D**). Moreover, increases in stimulation intensity from 0.2 to 2 mA induced a reproducible significant fMRI signal increase in the contralateral somatosensory cortex (**Figure S2**). Because this stimulation intensity ramping protocol was used to successfully identify the neural substrate under forepaw stimulation, the same protocol should be suitable to assess the substrates under the LV or RV stimulation.

### Validation of fMRI Signals under LV or RV Stimulation

The physiological parameters measured during and after fMRI scanning under LV or RV stimulation (**Table 1**) were comparable to those observed in the previous study [12] and thus should be suitable for the evaluation of fMRI signals in the present study. Although the pCO_2_ and pH values tended to be higher in LV stimulation than in RV stimulation, fMRI signals are entirely independent of pCO_2_ and pH values within the reported range of values [12].

**Table 1:** Physiological variables during fMRI scanning.

	LV stim	RV stim	P value
BW(g)	368±5	368±10	0.991
mABP(mmHg)	101±6	110±4	0.201
pH	7.40±0.02	7.44±0.01	0.064
pO_2_(mmHg)	143±7	150±4	0.471
pCO_2_(mmHg)	34.9±1.9	29.5±0.9	0.013
RT(°C)	36.8±0.5	37.0±0.1	0.229

All values are expressed as the mean±SEM. BW, body weight; fMRI, functional magnetic resonance imaging; LV, left ventricular; mABP, mean arterial blood pressure; RT, rectal temperature; RV, right ventricular.

**Figure 2:**
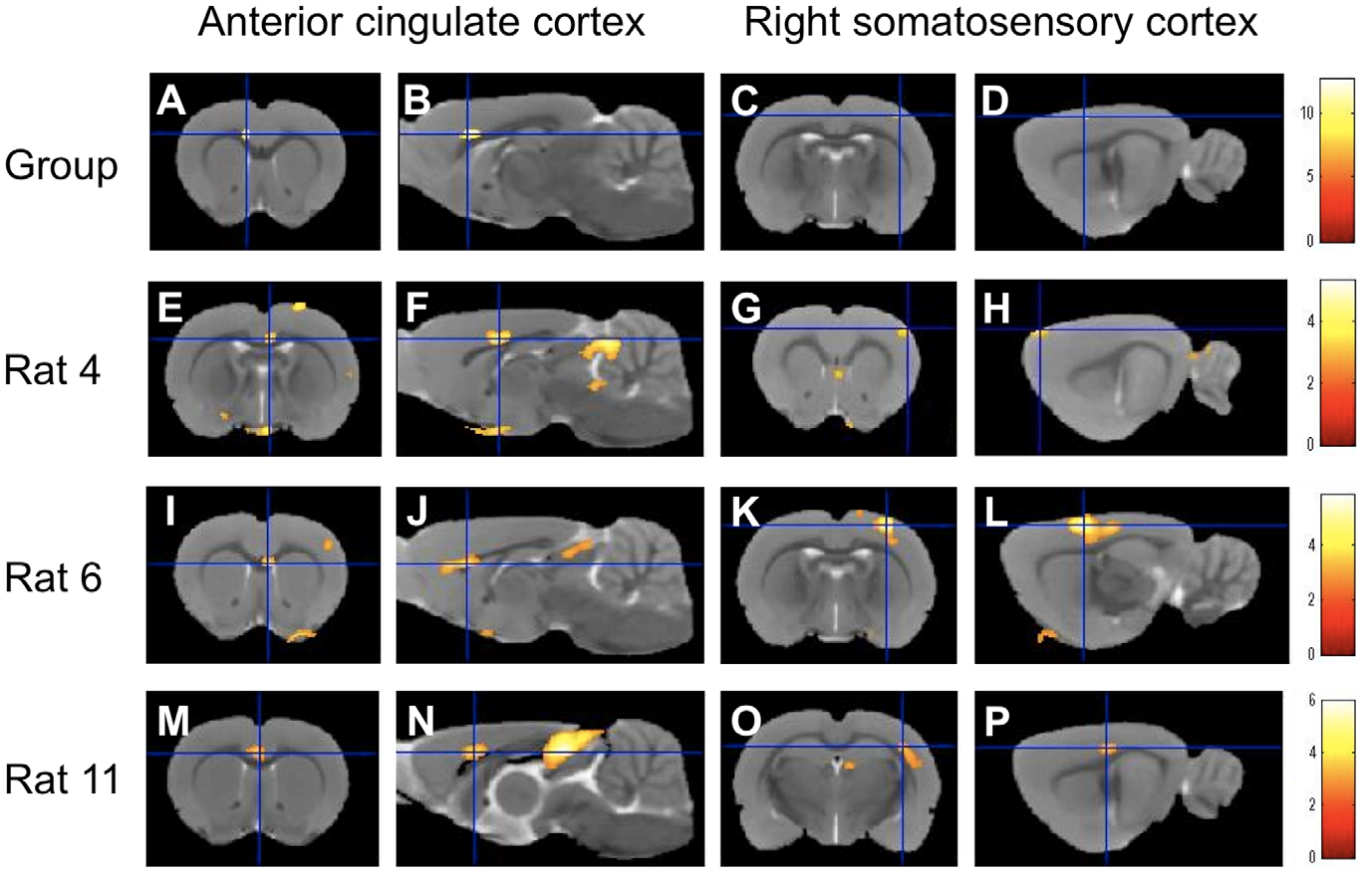
Brain activation under left ventricular stimulation. Stimulation of the left ventricle induced significant fMRI signal increases in the anterior cingulate cortex (**A, B**) and in the right somatosensory cortex (**C, D**), as measured in the second-level analysis (n = 12). Consistent with this group-level result, first-level analyses in three representative rats (rats 4, 6, and 11 quoted from **table 1**) demonstrated reproducible activations in the anterior cingulate cortex (**E, F, I, J, M, N**) and in the right somatosensory cortex (**G, H, K, L, O, P**). The results are displayed on the male Wistar rat template. The color calibration bars in each image represent critical t-score magnitudes for a threshold level of P<0.05 corrected for multiple comparisons using the false discovery rate (**A–D**) and P<0.005 uncorrected for multiple comparisons (**E–P**).

**Figure 3:**
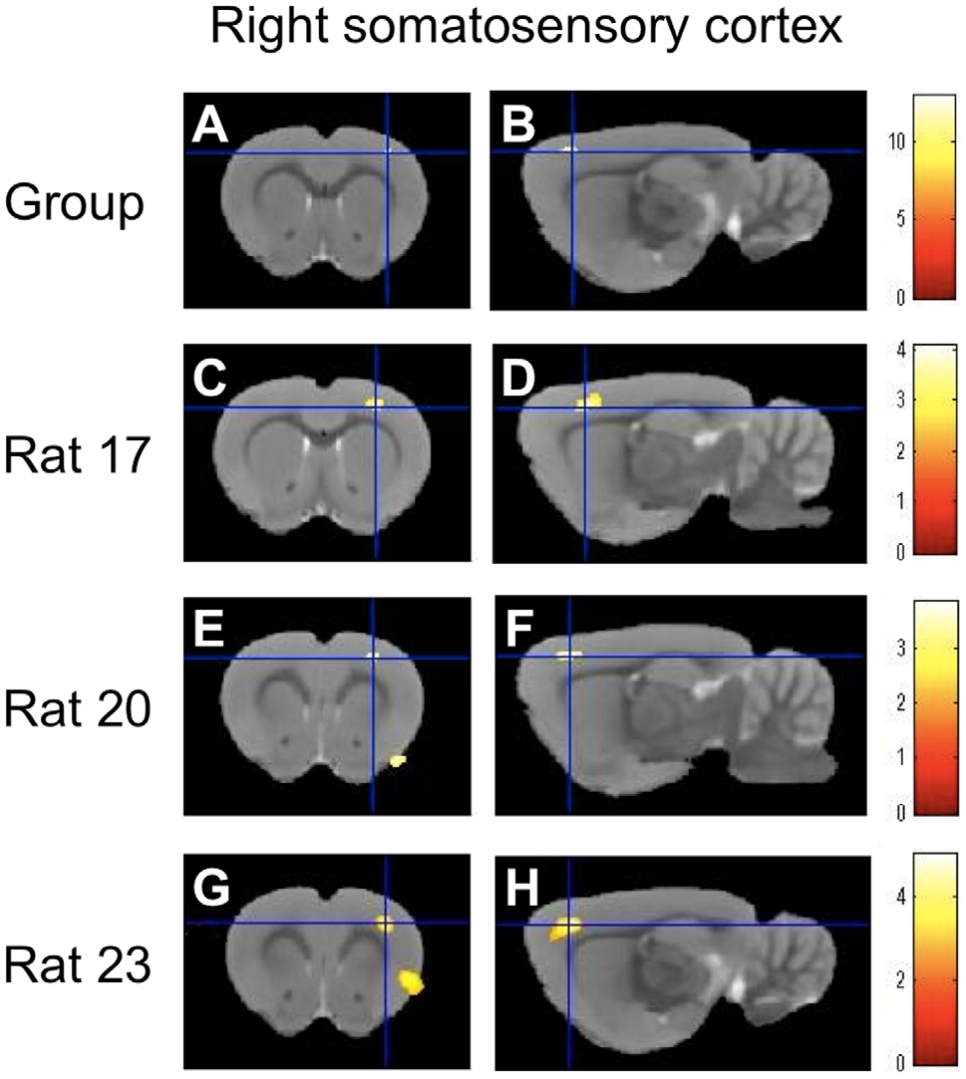
Brain activation under right ventricular stimulation. Stimulation of the right ventricle induced significant fMRI signal increases in the right somatosensory cortex (**A, B**), as measured in the second-level analysis (n = 12). Consistent with this group-level result, first-level analyses in three representative rats (rats 17, 20, and 23 quoted from **table 2**) demonstrated reproducible activations in the right somatosensory cortex (**C–H**). The results are displayed on the male Wistar rat template. The color calibration bars in each image represent critical t-score magnitudes for a threshold level of P<0.05 corrected for multiple comparisons using the false discovery rate (**A, B**) and P<0.005 uncorrected for multiple comparisons (**C–H**).

**Figure 4:**
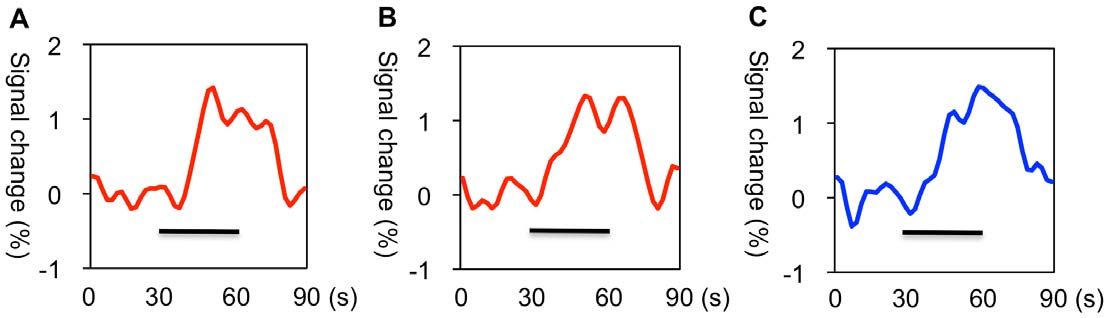
Temporal profiles of fMRI signals under left (LV) and right ventricular (RV) stimulation. Temporal profiles showing the averaged fMRI signal intensity changes of the anterior cingulate cortex (**A**, n = 10) and the right somatosensory cortex (**B**, n = 11) in response to LV stimulation (red lines) and the right somatosensory cortex (**C**, n = 11) in response to RV stimulation (blue line). The black bars represent the duration of the 2-mA stimulation.

In the second-level analysis (n = 12 each), LV stimulation caused significant fMRI signal increases in the anterior cingulate cortex and the right somatosensory cortex (**Figure 2** **A–D,** **table 2**), whereas RV stimulation caused a significant increase in the right somatosensory cortex but no significant activation in the anterior cingulate cortex (**Figure 3** **A, B,** **Table 3**). Activation of the right somatosensory cortex under cardiac stimulation is consistent with the left-sided pain perception during myocardial ischemia. No brain region was significantly deactivated during either LV or RV stimulation. Data from individual rats demonstrated the reproducibility of the activation in the anterior cingulate cortex and the right somatosensory cortex under LV stimulation (**Figure 2** **E–P,** **Table 2**) as well as in the right somatosensory cortex under RV stimulation (**Figure 3** **C–H,** **Table 3**). Significant activation was identified in 10 rats in the anterior cingulate cortex and 11 rats in the right somatosensory cortex under LV stimulation (**Table 2**). Significant activation of the right somatosensory cortex was observed in 11 rats under RV stimulation (**Table 3**). Temporal profiles showed that the fMRI signal increases induced by LV or RV stimulation correlated with the duration of the 2-mA stimulation (**Figure 4**). These fMRI results lead to the conclusion that the anterior cingulate cortex and right somatosensory cortex are the neural substrates under LV stimulation and the right somatosensory cortex is the neural substrate under RV stimulation.

**Table 2:** Brain activation under left ventricular stimulation.

	Anterior cingulate cortex			Right somatosensory cortex		
	Coordinates(x, y, z)	Zscore	Voxelsin cluster	Coordinates(x, y, z)	Zscore	Voxelsin cluster
Group	(–0.96, –3.14, 1.56)	5.39	222	(4.56, –1.94, –1.08)	5.12	61
Rat 1				(5.28, –2.78, 1.80)	3.16	100
Rat 2	(–0.36, –2.3, 1.92)	2.82	27	(5.4, –2.54, –0.84)	2.87	49
Rat 3	(1.68, –3.38, 0.84)	3.12	58	(4.80, –2.66, 3.00)	3.31	139
Rat 4	(0.60, –3.02, –0.72)	3.92	457	(5.04, –2.42, 1.92)	3.56	459
Rat 5	(–0.96, –3.5, 0.24)	2.95	94	(4.68, –2.42, –1.32)	2.77	23
Rat 6	(0.60, –4.22, 1.32)	4.03	833	(3.60, –1.58, –0.96)	5.28	3365
Rat 7	(0, –1.58, 1.32)	5.30	2408	(4.68, –3.14, 1.68)	3.20	336
Rat 8				(3.84, –2.06, –0.60)	3.17	470
Rat 9	(–0.84, –3.02, 0.12)	4.06	888	(5.64, –5.18, –2.16)	2.87	58
Rat 10	(0.36, –3.14, 1.56)	4.80	4320			
Rat 11	(0.12, –3.26, 0.84)	3.81	665	(4.68, –2.9, –2.88)	3.51	699
Rat 12	(0.60, –2.06, 1.44)	3.53	282	(4.92, –3.62, –2.04)	3.07	264

Coordinates are relative to bregma in the right-left (x), superior-inferior (y), and anterior-posterior (z) directions (mm). Voxels in each cluster are expressed as the number of voxels exceeding the threshold of P<0.05 corrected for multiple comparisons using the false discovery rate in the second-level analysis (Group) and P<0.005 uncorrected for multiple comparisons in the first-level analyses of rats 1–12. Blanks indicate that no voxels exceeded the significance threshold in the anterior cingulate cortex or in the right somatosensory cortex.

**Table 3:** Brain activation under right ventricular stimulation.

	Right somatosensory cortex		
	Coordinates(x, y, z)	Zscore	Voxelsin cluster
Group	(3.72, –2.3, 1.68)	5.44	77
Rat 13	(2.64, –1.22, –1.08)	4.64	1583
Rat 14	(4.44, –2.06, 0.24)	3.97	1128
Rat 15	(4.08, –2.3, 2.64)	2.72	23
Rat 16	(4.8, –2.06, 2.64)	2.86	116
Rat 17	(3.12, –2.42, 1.2)	3.62	487
Rat 18	(3.84, –2.54, 3.36)	4.63	7934
Rat 19	(3.72, –2.78, 1.92)	2.74	22
Rat 20	(3, –2.3, 2.04)	3.37	194
Rat 21	(3.36, –1.7, 2.64)	2.62	4
Rat 22	(5.16, –3.02, 0)	3.01	240
Rat 23	(3.6, –2.9, 2.16)	4.01	579
Rat 24			

Coordinates are relative to bregma in the right-left (x), superior-inferior (y), and anterior-posterior (z) directions (mm). Voxels in each cluster are expressed as the number of voxels exceeding the threshold of P<0.05 corrected for multiple comparisons using the false discovery rate in the second-level analysis (Group) and P<0.005 uncorrected for multiple comparisons in the first-level analyses of rats 13–24. Blanks indicate that no voxels exceeded the significance threshold in the right somatosensory cortex.

### Different Brain Activation under Left and Right Ventricular Stimulation

**Figure 5:**
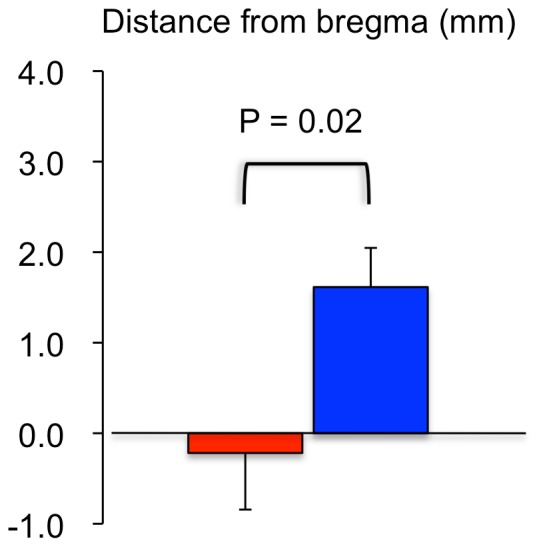
Anterior-posterior distances in the right somatosensory activation under left (LV) and right ventricular (RV) stimulation. Anterior-posterior distances from bregma in the right somatosensory activation focuses were averaged in 11 rats with LV stimulation (red bar) and 11 rats with RV stimulation (blue bar).

In the second-level analysis, significant activation of the anterior cingulate cortex was observed only in response to LV stimulation but not in response to RV stimulation. Moreover, the right somatosensory activation induced by RV stimulation was observed more anteriorly at 1.68 mm from bregma compared to the activation induced by LV stimulation at –1.08 mm from bregma (**Figure 2** **C,** **Figure 3** **A,** **Table 2** **,** **3**). To support the second-level analysis, data from individual rats demonstrated that the foci of the right somatosensory activation induced by RV stimulation were located in significantly more anterior regions (1.61±0.43 mm from the bregma) relative to those induced by LV stimulation (–0.22±0.63 mm from the bregma) (P = 0.02, **Figure 5**). These fMRI results support the hypothesis that fMRI signal changes in the different brain regions are observed under LV and RV stimulation.

## Discussion

In the present study, we used fMRI to investigate the brain regions activated in response to LV or RV stimulation in rats. LV or RV stimulation was performed with step-wise increases in stimulation intensity from 0.2 to 2 mA, which did not induce sudden or considerable hemodynamic changes, and caused an fMRI signal increase in the contralateral somatosensory cortex in the forepaw. To support the hypothesis, increased fMRI signals in the different brain regions were observed in response to LV and RV stimulation respectively. The brain regions activated by LV stimulation were the anterior cingulate cortex and the right somatosensory cortex, whereas RV stimulation activated the right somatosensory cortex, which was identified more anteriorly relative to LV stimulation but did not activate the anterior cingulate cortex. To our knowledge, this is the first study to show the different brain processes resulting from stimulation of different areas of the heart, such as the LV and RV.

### Brain Regions under LV or RV Stimulation

In the present study, fMRI signal increases in response to the LV or RV stimulation were detected in the anterior cingulate cortex and right somatosensory cortex. These results were consistent with the past study that Fos expression of the cingulate cortex and somatosensory cortex was increased in response to pericardial administration of bradykinin or capsaicin [35]. The anterior cingulate cortex and somatosensory cortex were most likely activated via the neuronal pathways of cardiac afferent nerves. The cell bodies of cardiac afferent nerves are present in the dorsal root and nodose ganglia [36]. Cardiac afferent nerves to nodose ganglia innervate the solitary nucleus [36]. Information from cardiac afferent nerves is then transmitted to the superficial and deep laminae of the dorsal horn in the spinal cord through dorsal root ganglia and the solitary nucleus [36], [37]. Ascending projections of the superficial and deep laminae mainly reach the lateral thalamus, medial thalamus, parabrachial nucleus and periaqueductal gray in rats [37]–[39]. The solitary nucleus and parabrachial nucleus send afferent projections to the periaqueductal grey in rats [37], [40]. These subcortical neural networks may relay information from cardiac afferent nerves to the cortical structures identified in the present study.

The anterior cingulate cortex was activated in response to LV stimulation in the present study. Dobutamine-induced myocardial ischemia has also been shown to activate the anterior cingulate cortex in humans [41]–[44]. Information from cardiac afferent nerves can be transmitted to the anterior cingulate cortex through the medial thalamus and periaqueductal gray in rats [45]–[48]. The anterior cingulate cortex is the motor center of the limbic system and is responsible for emotional arousal in humans [49]–[51]. Similar to humans, the anterior cingulate cortex is involved in emotional behaviors in rats [51], [52]. In accordance with these findings, the activation of the anterior cingulate cortex that we observed in the present study may be associated with emotional processing in rats during LV stimulation. The activation of the right somatosensory cortex was evoked by LV or RV stimulation. This activation of the right somatosensory cortex is consistent with the left-sided pain perception during myocardial ischemia. Information from cardiac afferent nerves can be transmitted to the somatosensory cortex through the lateral thalamus [36]. Convergent inputs from cardiac and somatosensory afferent nerves to common neurons in C1–Th9 in the spinal cord provide an explanation for the referred somatic pain that can be associated with myocardial ischemia [36]. Thus, the activation of the right somatosensory cortex may be associated with sensory discrimination in rats during the cardiac electrical stimulation.

### Different Brain Activation under LV and RV Stimulation

The different brain activations induced by LV and RV stimulation can be explained by the different innervations patterns of cardiac afferent nerves. The activation of the anterior cingulate cortex was observed under LV stimulation but not RV stimulation. There are two explanations for the lack of activation in the anterior cingulate cortex in response to RV stimulation. First, the RV is more sparsely innervated with cardiac afferent nerves than the LV [2]; therefore, a stimulation intensity greater than 2 mA may be necessary for the activation of the anterior cingulate cortex by RV stimulation. The second explanation is that not the neuronal pathway of cardiac afferent nerves to nodose ganglia (vagal afferents) but the pathway to dorsal root ganglia (sympathetic afferents) project to the anterior cingulate cortex. The anterior cingulate cortex is associated with sympathetic arousal in humans and rats [50], [53]–[55]. Sympathetic afferents are associated with sympathetic arousal, whereas decreases in blood pressure and heart rate were observed during stimulation of vagal afferents [1], [56]. Actually, sympathetic arousal mainly occurs in patients with myocardial ischemia of the anterior wall, which is predominantly innervated by sympathetic afferents [1], [3], [4]. In contrast, hypotension and bradycardia are often observed in patients with RV myocardial ischemia, where cardiac vagal afferents predominate [2]. Therefore, activation of the anterior cingulate cortex under LV stimulation may be associated with the stimulation of sympathetic afferents, whereas the lack of activation in the anterior cingulate cortex in response to RV stimulation may be explained by the poor innervation of sympathetic afferents in the present study.

The activation of the right somatosensory cortex was identified more anteriorly under RV stimulation than under LV stimulation. A possible explanation for these different somatosensory activations is that sympathetic and vagal afferents would project to different regions of the somatosensory cortex. The stimulation of vagal afferent fibers excites neurons in the C1–C3 segments, which have somatic receptive fields that are found mainly in the neck, jaw, ear and upper arm [36], [57]. Inputs from sympathetic afferents also excite neurons in the C1–C3 segments but seem to play a subordinate role because vagal stimulation markedly increases cell activity with C-fiber input more often than the stimulation of sympathetic afferents [36]. Moreover, jaw or neck pain induced by myocardial ischemia was reported in patients who have coronary artery disease and are quadriplegic because of a lower cervical segment injury, which would eliminate possible inputs to the upper cervical segments from sympathetic afferents [36]. This evidence indicates the different somatosensory projections between sympathetic and vagal afferents and supports the different patterns of somatosensory activation under LV and RV stimulation in the present study.

### Study Limitations

Several limitations should be mentioned for the present study. First, electrical stimulation was used to stimulate the heart in the present study. It is not possible to identify precisely which fibers are stimulated by electrical stimulation [58]. Therefore, our results should be re-evaluated using more physiological stimulus such as coronary ligation of the left or right coronary artery. Second, fMRI techniques in the present study cannot discriminate between afferent and efferent or vagal and sympathetic neural activity. Microneurographic experiments may be useful to separate these nerve traffic [59]. Third, the present study was performed using α-chloralose anesthesia. This anesthesia is commonly used for rat fMRI studies because it causes minimal cardiovascular effects [60], and fMRI signals are well localized under this anesthesia compared to the signals observed using other anesthetics or recorded in the awake state [61]–[63]. However, α-chloralose, isoflurane and the awake state cause distinct effects on fMRI signal changes in response to gut stimulation in several brain regions [25]. Therefore, α-chloralose might influence the present findings of the entire functional response to the LV and RV stimulation. Fourth, the present study was performed in rats, not in humans. Craig [64] described neuroanatomical differences associated with visceral sensation between rats and humans. For instance, rats do not have a structure homologous to the anterior insula, which is crucial for subjective feelings of visceral sensation in humans. However, combined MRI and cardiac electrical stimulation is not currently considered safe for human scientific studies [9], [10]. Our results should be re-evaluated in humans with the research using positron emission tomography (PET) in conjunction with cardiac pacing devices.

In conclusion, the present study provides the first evidence for different brain activation in response to LV and RV stimulation. These resulting differences in brain processes may be associated with different clinical manifestations between anterior wall myocardial ischemia and inferoposterior wall and/or RV myocardial ischemia.

## Materials and Methods

### Animal Preparation

All procedures and protocols were performed in agreement with the policies established by the Animal Care Committee at Tohoku University, Sendai, Japan (approved protocol number #22-351).

Animal experiments were performed in 26 male Wistar rats (9–11 weeks old, 367.5±4.9 g; Charles River, Yokohama, Japan). The animals were anesthetized with isoflurane prior to insertion of polyethylene catheters into the femoral artery and vein for examining physiological variables and delivering drugs systemically. For forepaw electrical stimulation, a pair of small needle electrodes (NE-224S, Nihon Kohden, Tokyo, Japan) was inserted under the skin of the right forepaw (n = 2). For LV or RV stimulation, a platinum bipolar catheter (FTS-1913A-1018, Scisense, Ontario, Canada) was introduced through the right carotid artery or right jugular vein to the LV or RV (n = 12 each). Platinum devices are highly MRI-compatible and induce minimal imaging artifacts [65]; the typical direct current resistance to the electrode was 18 Ω according to the manufacturer. Animal preparations for fMRI recordings have been previously described [12]. In brief, the animals were orally intubated for artificial ventilation, inhalation of isoflurane was discontinued, and α-chloralose (80 mg/kg) was administered intravenously. The animals were placed in a prone position on an MRI bed with a bite bar and were mechanically ventilated at a respiration rate of 60±1 breaths/min using a ventilator (SAR-830/AP, CWE Inc., Ardmore, PA, USA). Rectal temperature was continuously monitored with an MRI-compatible temperature probe (Model 1025, SA Instruments, Stony Brook, NY, USA) and was maintained at 37.0±1.0°C during the experiment using a water-circulating pad. A bolus injection of α-chloralose (20 mg/kg) and pancuronium (2 mg/kg) was administered 30 min after the first administration of α-chloralose, followed by continuous administration of α-chloralose (26.7 mg/kg/hr) and pancuronium (2 mg/kg/hr) until the end of the experiment.

### Forepaw and Cardiac Electrical Stimulation

Electrical pulses were produced by a generator (SEN-3401, Nihon Kohden, Tokyo, Japan) and an isolator (SS203-J, Nihon Kohden, Tokyo, Japan) and were selectively added to the forepaw between the two needle electrodes or to the heart between the bipolar electrodes at a distance of 1 mm from the tip of the catheter. A cardiac stimulation catheter was placed at the LV or RV pacing position using echocardiography (Vevo2100, VisualSonics, Ontario, Canada). Chest wall twitching did not occur during 2 mA cardiac pacing prior to pancuronium administration. Cardiac or forepaw electrical stimulation was performed by increasing the electrical stimulation intensity from 0.2 to 2 mA.

### Experimental Protocols

Brain activation during the cardiac or forepaw electrical simulation was investigated by fMRI scanning using a block-design stimulation paradigm. We modified the fMRI protocols of the cardiac electrical stimulation based on our previous study in which we obtained CEP recordings in response to cardiac electrical stimulation [7]. For habituation of the pacing-induced hemodynamic effects, fMRI scanning was performed 30 min after starting LV or RV pacing with 0.2 mA. The pacing frequency and pulse width were fixed at 9 Hz and 0.3 msec, respectively. During LV or RV pacing, a block-design stimulation paradigm consisting of 10 blocks was employed; each block was comprised of 2 mA stimulation for 30 sec, followed by 0.2 mA stimulation for 60 sec. The same paradigm was applied to fMRI scanning during the forepaw stimulation.

### Physiological Parameter Monitoring

Mean arterial blood pressure (mABP), HR, and rectal temperature were simultaneously monitored during each fMRI scan, and arterial blood was sampled for blood gas analysis after each fMRI scan. Blood pressure waves transmitted from the arterial polyethylene catheter were digitized by a pressure transducer (DTXPlus^TM^, BD, Franklin Lakes, NJ, USA), amplified by an MEG-6108 amplifier (Nihon Kohden, Tokyo, Japan), and analyzed with a PowerLab/16SP and LabChart 6 device (ADInstruments, Colorado Springs, CO, USA). Blood gas analysis was performed with a Rapidlab 248 system (Siemens, Munich, Germany). Physiological monitoring is important for evaluating fMRI signals because the coupling between fMRI signals and basal neural activity is significantly affected by physiological parameters such as HR [12].

### MRI Recordings

All MRI data were acquired using a 7T Bruker PharmaScan system (Bruker Biospin, Ettlingen, Germany) with a 38-mm diameter bird-cage coil. Global magnetic field shimming was performed inside the core prior to all MRI experiments and was later evaluated within a region of interest (ROI) using a point resolved spectroscopy protocol [12]. The line width (full width at half maximum) at the end of the shimming procedure ranged from 12 to 20 Hz in the ROI (approximately 300 µl). T2-weighted anatomical images were obtained for image normalization to the rat brain atlas template [66] using the following 2D-RARE sequence: TR = 4600 msec, TEeff = 30 msec, RARE factor = 4, SBW = 100 kHz, flip angle = 90^o^, FOV = 32×32 mm^2^, matrix size = 256×256, voxel size = 125×125 µm^2^, number of slices = 54, slice thickness = 0.5 mm, slice gap = 0 mm, and number of averages = 10. fMRI signals were obtained using gradient-echo echo-planer imaging (GE-EPI) with the following parameters: TR = 2000 msec, TE = 15 msec, SBW = 250 kHz, flip angle = 30°, FOV = 25×14 mm^2^, matrix size = 125×70, voxel size = 200×200 µm^2^, number of slices = 18, slice thickness = 1.5 mm, slice gap = 0 mm, number of volumes = 480, and dummy scan number = 4.

### fMRI Data Analysis

The preprocessing procedures and fMRI data analyses were performed using Statistical Parametric Mapping software (SPM8, Welcome Department of Cognitive Neurology, London, UK) implemented in MATLAB (Mathworks Inc., Natick, MA, USA). The pre-processing procedures were performed as follows. First, the anatomical and EPI images were resized by a factor of 10 and were re-aligned and re-sliced to adjust for head movement. The EPI images were corrected for slice order acquisition. Second, re-aligned anatomical and EPI images were averaged to produce their mean images. The mean anatomical images were co-registered to the mean EPI image. Third, the co-registered anatomical images and re-aligned EPI images were roughly aligned with the Wistar rat template brain [66] and then the co-registered anatomical images were segmented into the three tissue classes by the unified segmentation approach [67] and the probabilistic maps associated with this template [66] after aligning the co-registered anatomical images and re-aligned EPI images with the template. Fourth, the re-aligned EPI images were spatially normalized into the template space using the parameterization of deformation fields in the previous segmentation. Finally, the normalized EPI images were smoothed using a Gaussian kernel with a full width at half maximum of 0.8 mm in the x, y, and z-axes.

First-level fMRI analysis was performed on the smoothed EPI images from individual animals, and the second-level analysis combined the results of the first-level analyses across individuals to investigate group effects. The resulting t-statistic parametric maps from the forepaw and from either LV or RV stimulation were overlaid onto the Wistar rat template. To evaluate fMRI signal changes during the forepaw electrical stimulation, first-level analysis results were used for each rat. In the analysis of LV or RV stimulation, brain regions with significant fMRI signal changes were identified using the second-level analysis and were subsequently confirmed by individual data, which demonstrated reproducibility and temporal profiles of fMRI signal changes. To evaluate the temporal profiles of fMRI signal changes, we extracted a time course from the voxel with the highest t-score for each condition in each individual rat. The time courses were low-pass filtered (6th order Butterworth filter, <0.1 Hz) and averaged for each condition. The anterior-posterior focuses of the right somatosensory cortex under the LV stimulation were compared with those under the LV stimulation using data from the first-level analyses.

### Statistical Analysis

The physiological parameters collected during and after the fMRI scans and the anterior-posterior focuses in response to LV or RV stimulation were expressed as the mean ± standard error of mean (SEM) at a significance level of P<0.05. For the first-level analyses of the forepaw electrical stimulation, a significance level was set at P<0.05 corrected for multiple comparisons using the family-wise error (FWE corrected). For the first-level and second-level analyses of LV and RV stimulation, the significance levels were set at P<0.005 uncorrected for multiple comparisons (uncorrected) and at P<0.05 corrected for multiple comparisons using the false discovery rate (FDR corrected) more than 20 pixels, respectively.

## Supporting Information

Figure S1 Representative pictures of the hemodynamic effects of cardiac electrical stimulation. Left ventricular stimulation was performed by starting cardiac pacing with 0.2 mA **(A, C)** and increasing the pacing intensity from 0.2 to 2 mA (**B, D**). These hemodynamic effects of the left ventricular stimulation were similar to those of right ventricular stimulation.
(TIF)

Figure S2 Brain activation under right forepaw stimulation. Increasing the electrical stimulation intensity from 0.2 to 2 mA on the right forepaw reproducibly induced significant fMRI signal increases in the left somatosensory cortex (**A, B**). The results are displayed on the male Wistar rat template. The color calibration bars in each image represent critical t-score magnitudes for a threshold level of P<0.05 corrected for multiple comparisons using the family-wise error. The coordinates of fMRI signal increases are relative to bregma in the right-left (x), superior-inferior (y), and anterior-posterior (z) directions (mm). In **A** and **B**, (x, y, z)  =  (–3.72, –1.58, 0.96) and (–3.24, –2.42, 0.72), respectively.
(TIF)
