# Supplementary figures and images for: Different Brain Activation under Left and Right Ventricular Stimulation: An fMRI Study in Anesthetized Rats
Source: PLoS One. 2013 Feb 22;8(2):e56990. doi: 10.1371/journal.pone.0056990 (PMC3579932; doi:10.1371/journal.pone.0056990)

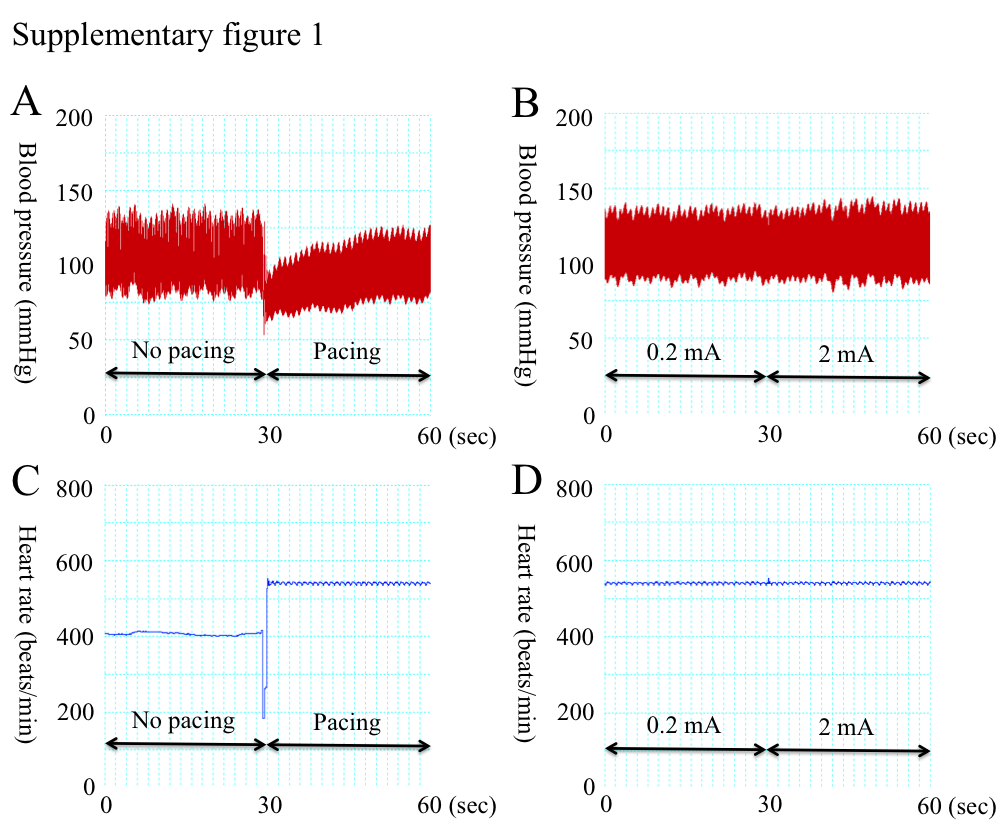

Supplement: Figure S1 — Representative pictures of the hemodynamic effects of cardiac electrical stimulation. Left ventricular stimulation was performed by starting cardiac pacing with 0.2 mA (A, C) and increasing the pacing intensity from 0.2 to 2 mA (B, D). These hemodynamic effects of the left ventricular stimulation were similar to those of right ventricular stimulation. (TIF) [file pone.0056990.s001.tif]

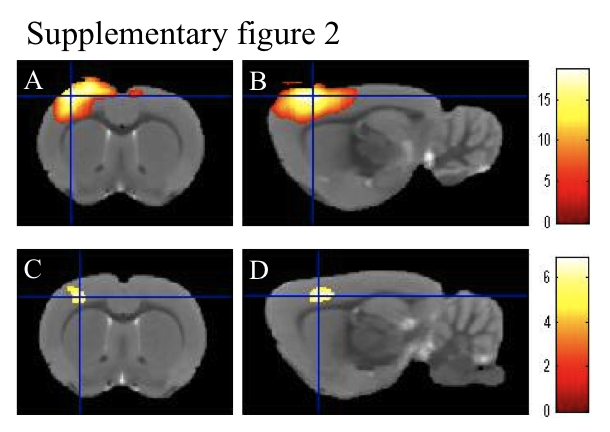

Supplement: Figure S2 — Brain activation under right forepaw stimulation. Increasing the electrical stimulation intensity from 0.2 to 2 mA on the right forepaw reproducibly induced significant fMRI signal increases in the left somatosensory cortex (A, B). The results are displayed on the male Wistar rat template. The color calibration bars in each image represent critical t-score magnitudes for a threshold level of P<0.05 corrected for multiple comparisons using the family-wise error. The coordinates of fMRI signal increases are relative to bregma in the right-left (x), superior-inferior (y), and anterior-posterior (z) directions (mm). In A and B, (x, y, z) = (–3.72, –1.58, 0.96) and (–3.24, –2.42, 0.72), respectively. (TIF) [file pone.0056990.s002.tif]
